# Containment of sulfate in leachate as gypsum (CaSO_4_·2H_2_O) mineral formation in bio-cemented sand via enzyme-induced carbonate precipitation

**DOI:** 10.1038/s41598-023-37772-z

**Published:** 2023-07-06

**Authors:** Junghoon Kim, Daehyun Kim, Tae Sup Yun

**Affiliations:** 1 Department of Civil and Environmental Engineering, Yonsei University, Yonsei-ro 50, Seodaemun-gu, Seoul, 03722 Republic of Korea; 2 Department of Civil and Environmental Engineering, Hiroshima University, 1‑4‑1 Kagamiyama, Higashi‑Hiroshima, Hiroshima, 739‑8527 Japan

**Keywords:** Biomineralization, Sustainability, Environmental sciences

## Abstract

Enzymatically induced carbonate precipitation (EICP) using urea hydrolysis is a well-known bio-cementation process that not only promotes the precipitation of calcium carbonate (CaCO_3_) but can provide excess calcium cations for further reaction depending on the substrate constituents and reaction stage. This study presents the EICP recipe to contain sulfate ions in landfill leachate sufficiently using remaining calcium cations and a series of tests were conducted to validate its ability to retain sulfates. The reaction rate for 1 M CaCl_2_ and 1.5 M urea was identified by controlling the purified urease content and the curing time of the EICP process. The results showed that 0.3 g/L of purified urease produced 46% CaCO_3_ and reduced sulfate ions by 77% after 3 days of curing. The shear stiffness in EICP-treated sand was enhanced 13 times by CaCO_3_ precipitation followed by 1.12 times increment due to subsequent precipitation of gypsum (CaSO_4_·2H_2_O) crystals implying sulfate containment. A cost-efficient EICP treatment using soybean crude urease instead of lab-grade purified urease exhibited lower sulfate removal efficiency (i.e., 18%) with only nominal formation of gypsum in the EICP-treated sand. The addition of gypsum powder was effective in increasing sulfate removal by 40% when soybean crude urease was used for EICP.

## Introduction

Landfilling remains one of the most popular strategies for the disposal of Municipal Solid Waste (MSW) made attractive by the relatively low costs for construction and maintenance^1–3^. Despite these advantages, landfilling inherently causes environmental issues during its operation; leachate containing dissolved organic compounds, heavy metals, xenobiotic organic compounds, and inorganic macro components are continuously generated and seepage of these by-products contaminates soil and groundwater^4^. In particular, sulfate (${\text{SO}}_{4}^{2 - }$), a major component of MSW landfill leachate, is persistently present in high concentrations (250–1000 mg/L)^5^ worldwide (Table 1), which can adversely affect the natural sulfur cycle and human health^6,7^.

**Table 1:** Sulfate concentration ranges in leachate from landfill sites in different locations worldwide.

Country	Landfill Site	${\text{SO}}_{4}^{2 - }$ conc. (mg/L)	References
USA	–	500–2000	Farquhar^70^
Greece	Thessaloniki	400–2500	Tatsi et al.^71^
Ireland	–	7.2–1950	Brennan et al.^72^
Poland	Wysieka	98–374	Kulikowska & Klimiuk^73^
China	Jiangmen	50–400	Li et al.^74^
India	Chandigarh, Panchkula	364–2126	Negi et al.^75^
Indonesia	Gresik Regency	539.3	Bagastyo et al.^76^
Korea	Incheon	33–6286	
Malaysia	Kuala Lumpur	497	Emmanuel et al.^77^
Egypt	Alexandria	298–720	Abd El-Salam & Abu-Zuid^78^
Nigeria	Effurun	170–750	Asibor et al.^79^

For the removal of ${\text{SO}}_{4}^{2 - }$ in the landfill leachate, several methods have been reported. Coagulating and precipitating ${\text{SO}}_{4}^{2 - }$ by providing metal cations such as ferric, aluminum, and calcium ions, or lime (CaO) can reduce the concentration of ${\text{SO}}_{4}^{2 - }$ significantly^8–11^. However, these can affect the ambient environment by increasing the local concentration of specific ions in liquid and require the adjustment of pH with disposing of the bulky sludges generated^9,12,13^. Electrocoagulation using aluminum electrodes showed high ${\text{SO}}_{4}^{2 - }$ removal capacity of up to 95%^14^ with drawbacks such as high energy consumption by the formation of oxide film^15^.

Traditional landfill liners include compacted clay liners, which are designed to minimize the seeping of leachate into the groundwater, tending to adsorb ${\text{SO}}_{4}^{2 - }$ ions by ligand exchange and retention in the diffuse double layer^16,17^. However, previous studies have shown that a negligible amount of ${\text{SO}}_{4}^{2 - }$ was adsorbed by the clay minerals, which implies there is a need to develop novel MSW landfill liners that efficiently contain ${\text{SO}}_{4}^{2 - }$^16,18^.

Biologically-induced ground improvement techniques have recently attracted increased interest to modify hydro-physical properties of soil systems by the formation of biomineral^19–23^, biogas^24–27^, biofilm^28–30^, or biopolymer^31–33^. These biological processes have derived shear strength enhancement^19,20,22^, permeability control^31,34,35^, and mitigation of soil liquefaction potential^25,27,36^. Among them, bio-cementation representing binding particles through calcium carbonate (CaCO_3_) precipitation can be applied for slope stabilization^37^, dust suppression for wind erosion control^38–40^, concrete crack healing^41,42^, and heavy metal immobilization^43^. Also, some recent studies focus on capturing and storing carbon dioxide (CO_2_) by specific bacteria resulting in the mineralization of CaCO_3_ with calcium ions^44,45^ and reducing toxic by-products from urea hydrolysis through chemical precipitation with magnesium and biphosphate ions^46^. Mostly the research on bio-cementation has been concentrated on the formation of CaCO_3_ as an alternative to address environmental issues, and another approach related to the utilization of the calcium ions in cementation solution has almost not been studied.

This study proposes a passive strategy utilizing bio-cementation technique to contain ${\text{SO}}_{4}^{2 - }$ using calcium cations as the effective management and treatment of ${\text{SO}}_{4}^{2 - }$ in leachate passing through the liner system. For this purpose, Enzymatically Induced Carbonate Precipitation (EICP) technique was selected, and the CaCO_3_ precipitation reaction^21,47,48^ are as follows:

1 $${\text{CO}}\left( {{\text{NH}}_{2} } \right)_{2} + 3{\text{H}}_{2} {\text{O}}\to ^{{{\text{Urease}}}} 2{\text{NH}}_{4}^{ + } + {\text{HCO}}_{3}^{ - } + {\text{OH}}^{ - }$$

2 $${\text{HCO}}_{3}^{ - } \to {\text{CO}}_{3}^{2 - } + {\text{H}}^{ + }$$

3 $${\text{Ca}}^{2 + } + {\text{CO}}_{3}^{2 - } \to {\text{CaCO}}_{3} \left( \downarrow \right)$$

EICP not only forms CaCO_3_ crystals from hydrolyzed urea but depending on the precipitation stage or available elements, may also leave excess calcium cations. It is therefore hypothesized that in the presence of these excess calcium cations, ${\text{SO}}_{4}^{2 - }$ in leachate can also be mineralized into gypsum (CaSO_4_·2H_2_O), which is a solid compound that is thermodynamically stable at low temperatures (< 60 °C). This reaction can be described as follows:

4 $${\text{Ca}}^{2 + } + {\text{SO}}_{4}^{2 - } + 2{\text{H}}_{2} {\text{O}} \to {\text{CaSO}}_{4} \cdot 2{\text{H}}_{2} {\text{O}}\left( \downarrow \right)$$

**Figure 1:**
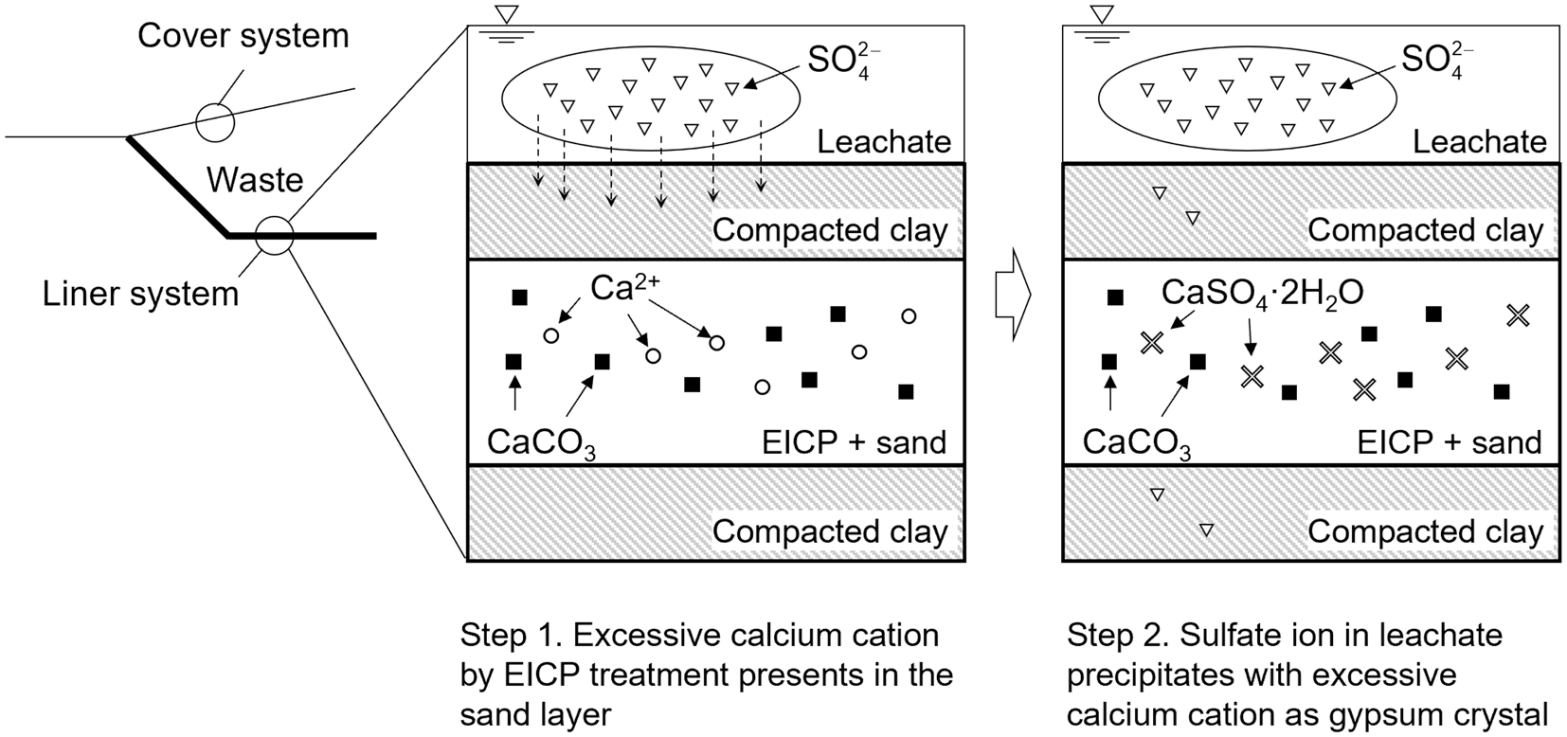
Schematical illustration of a proposed liner structure assisted by a partially EICP-treated sand layer. Step 1: Precipitation of CaCO_3_ crystal facilitates bonding between sand particles. Manipulation of EICP composition controls number of excess calcium cations remaining in the sand layer. Step 2: The ${\text{SO}}_{4}^{2 - }$ ions in the leachate react with pre-existing calcium cations, rapidly precipitating gypsum crystals in the sand layer which acts as a leachate reservoir with a relatively larger void space.

It is noted that the presence of calcium carbonate does not affect the precipitation of gypsum^49^. Given this hypothetical reaction sequence, the installment of a partially EICP-treated sand layer as a reservoir of dissolved calcium ions between compacted clay layers is proposed (Fig. 1). By targeting the ${\text{SO}}_{4}^{2 - }$ containment capacity within the EICP-treated sand layer, both EICP and gypsum formation were first balanced and optimized. The ${\text{SO}}_{4}^{2 - }$ captured by gypsum formation using the optimized EICP formula was confirmed by measurement of the shear wave velocity and visualization using Scanning Electron Microscopy (SEM). The effluent from the sand-layer model was analyzed to evaluate ${\text{SO}}_{4}^{2 - }$ removal efficiency. For cost-effectiveness, soybean crude urease was adopted as an alternative to purified urease, and the biocementating ability and ${\text{SO}}_{4}^{2 - }$ retention was tested with the same experimental procedure described above. In addition, the utilization of gypsum powder in soybean crude urease-triggered EICP technique was discussed in order to improve ${\text{SO}}_{4}^{2 - }$ removal efficiency.

This study proposes a novel application of bio-cementation technique via EICP for ${\text{SO}}_{4}^{2 - }$ containment in landfill leachate. The feasibility of a multi-functional liner system employing EICP technique was experimentally demonstrated. Based on the optimized recipe for EICP reaction, the proposed concept installing semi-EICP treated sand between two clay liners is expected to perform as a temporary reservoir for leachate and to provide excessive dissolved calcium cations which is a potential captor of ${\text{SO}}_{4}^{2 - }$.

## Materials and methods

### Sand materials

Jumunjin sand served as the interlayer base and possessed the following properties: a poorly graded sand (SP) with a specific gravity of 2.65, a uniformity coefficient (C_u_) of 1.94, a coefficient of gradation (C_c_) of 1.09, a mean grain size (D_50_) of 0.542 mm, a maximum void ratio (e_max_) of 0.897, a minimum void ratio (e_min_) of 0.6^50,51^.

### Determination of EICP recipe for CaCO_3_ formation and ${\text{SO}}_{4}^{2 - }$ removal: batch test

To maintain an excess of calcium cations in the EICP solution, the amount of produced CaCO_3_ was determined depending on the urea hydrolysis rate and curing time. A series of reaction tests with different urease concentrations and curing times were systematically conducted: (1) 0.1–0.9 g/L of lab-grade purified urease (extracted from jack bean, U1500, Sigma Aldrich, 40,150 U/g activity), and (2) curing time of 3, 14, and 72 days. The EICP solution was formulated using 1.5 M urea (U5378, Sigma Aldrich) and 1 M calcium chloride dihydrate (CaCl_2_∙2H_2_O, C3881, Sigma Aldrich). This urea-prevailing recipe with a 1.5:1 urea-calcium molar ratio was adopted from previous studies^47,50^, because although the EICP reaction theoretically requires calcium and urea at a 1:1 molar ratio and assumes the complete conversion of chemicals into CaCO_3_, full hydrolysis of the urea cannot be confirmed. One solution, 5 ml, consisting of urea-CaCl_2_∙2H_2_O with pH controller (1.5 × 10^−4^ M sodium hydroxide, NaOH), and another 5 ml of urease solution were prepared separately. Then, each solution was added to a 15 ml conical tube and mixed by softly shaking up and down 3 times. Curing took place at a room temperature of 25 °C. When the curing time specified by the experiment was over, the mass of precipitated CaCO_3_ minerals was measured using an acid digestion technique, following the protocol described in ASTM D4373-21^52^. The CaCO_3_ production rate was defined as the ratio of the mole of precipitated CaCO_3_ to the mole of theoretically producible maximum CaCO_3_ which was the same at the mole of the injected CaCl_2_ assuming full conversion of calcium cations to CaCO_3_.

**Figure 2:**
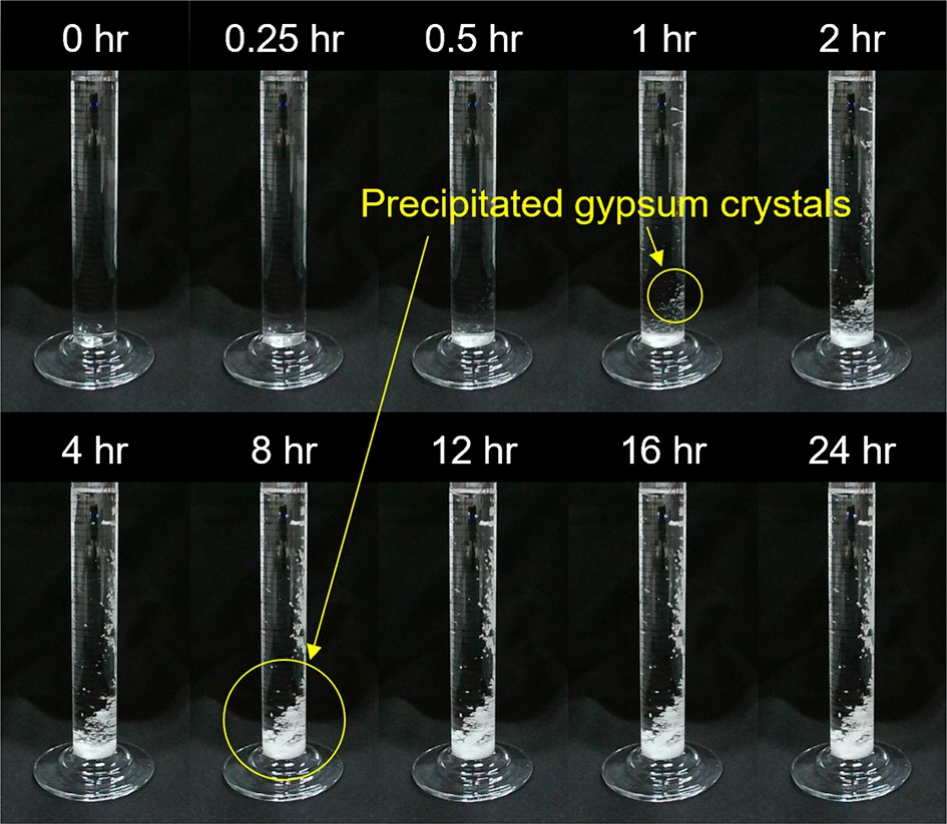
Precipitation process of calcium sulfate dihydrate (gypsum) with time. An aqueous solution contains 0.1 M of CaCl_2_∙2H_2_O and 0.1 M of Na_2_SO_4_∙10H_2_O. The precipitated crystal was visible after 1 h of reaction, production rate seemed to remain constant from 8 h of reaction.

The removal efficiency of ${\text{SO}}_{4}^{2 - }$ was confirmed after 3 days of curing based on pre-treated EICP samples with varying urease concentrations (0.1–0.9 g/L). An aliquot of 3 ml extracted from the supernatant separated with the sediment in 10 ml of EICP solution was mixed thoroughly with an equivalent volume of 0.1 M sodium sulfate (Na_2_SO_4_, 238597, Sigma Aldrich) solution in a conical tube by shaking up and down 3 times. In this study, a 0.1 M ${\text{SO}}_{4}^{2 - }$ solution prepared by mixing distilled water and Na_2_SO_4_ acted as the synthesized leachate and was used to diminish the influence of other ions typically present in MSW landfill leachate on gypsum formation. The mixture of EICP and 0.1 M ${\text{SO}}_{4}^{2 - }$ solution was allowed to react, the reaction time was determined from the optical observation of gypsum precipitation using time interval imaging (Fig. 2). After 24 h reaction, ${\text{SO}}_{4}^{2 - }$ concentration was measured using ion chromatography analysis (ICS-1100, Thermo fisher) of a 100 × diluted solution.

### Monitoring of shear wave velocity in sand layer during EICP reaction and ${\text{SO}}_{4}^{2 - }$ exposure

**Figure 3:**
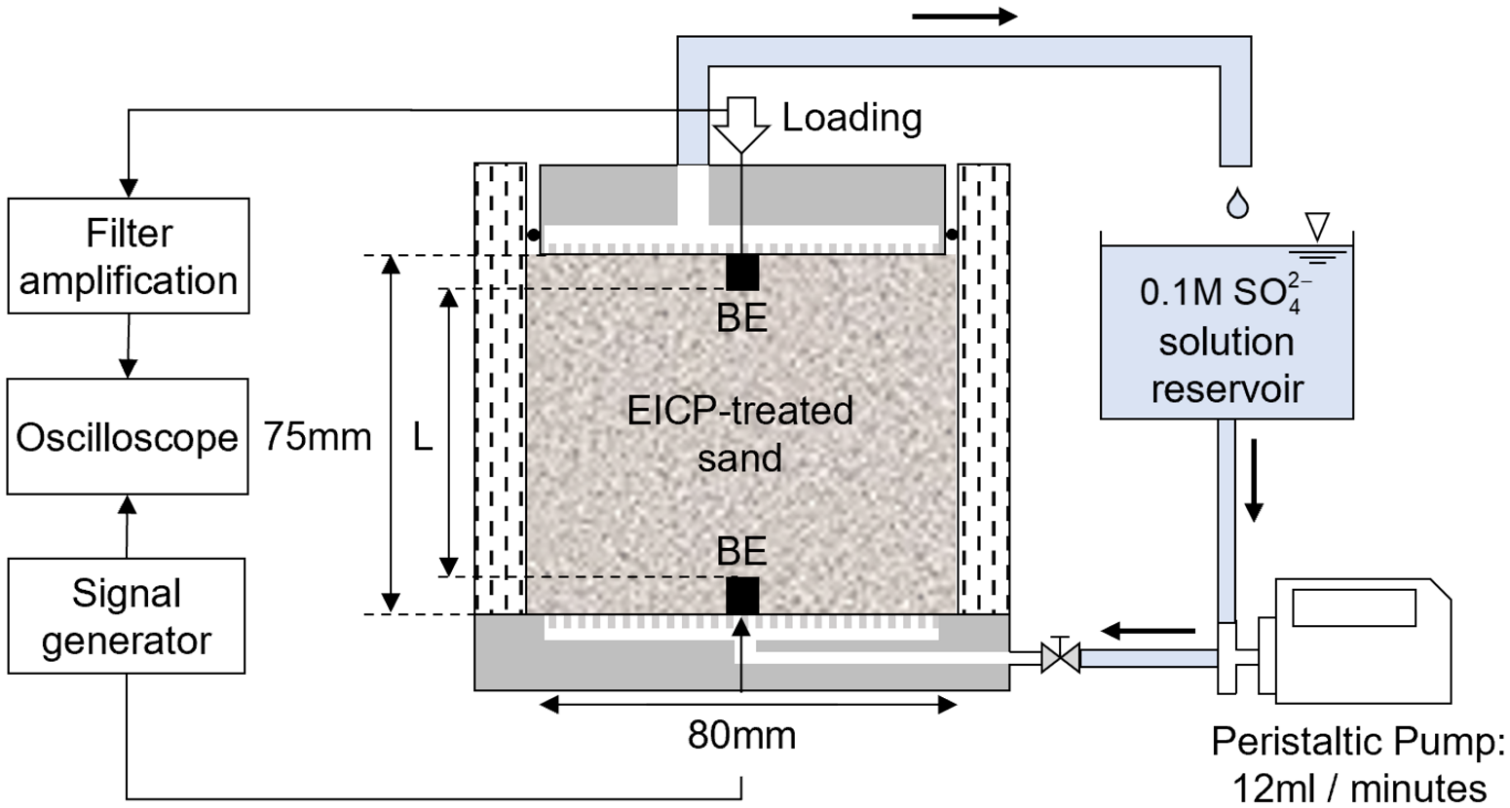
Experimental devices for measuring S-wave velocity during the EICP treatment and ${\text{SO}}_{4}^{2 - }$ exposure reaction.

The effect of CaCO_3_ and CaSO_4_·2H_2_O precipitation during the EICP treatment and SO_4_^2-^ exposure to the sand specimen on the shear stiffness was examined. A polycarbonate oedometer cell used was manufactured for measuring the shear (S-) wave velocity and cell details can be seen in Fig. 3. A pair of bender elements were installed on the top and bottom caps to generate and receive the S-wave pulses. A square wave signal at 50 Hz generated by a waveform generator (33220A, Agilent) was propagated by one bender element at the bottom, and the propagated wave was amplified and filtered using a band filter operating at frequencies between 100 Hz and 500 kHz (Model 3944, Krohn-hite). The received signal was captured and saved by an oscilloscope (DSO5014, Agilent).

The EICP treatment solution determined in “Determination of EICP recipe for CaCO_3_ formation and${\text{SO}}_{4}^{2 - }$removal: batch test” section was prepared and poured into the designed cell. 604 g of Jumunjin sand was instantly filled into the cell with EICP solution in three lifts. Each lift was gently tamped ensuring the sand reached 75 mm height precisely and the packing condition at 70% of the relative density. The volume of the EICP treatment solution corresponded to one pore volume of the 70% relative density sand, and the EICP reaction proceeded for 72 h under 10 kPa of vertical loading pressure. Then, 0.1 M ${\text{SO}}_{4}^{2 - }$ solution, corresponding to two pore volumes of the sand specimen, was circulated through circulation system at 12 mL/min by a peristaltic pump (BT-100CA, JIHPUMP) from the bottom to top for 26 min. The circulation rate was determined by assuming that the dissolved calcium cations were located mainly in the sand pores after circulation. The reaction with ${\text{SO}}_{4}^{2 - }$ solution continued for 24 h in the closed circulation system. The S-wave velocity was measured by selecting the first deflection point as the first arrival time-point and was monitored continuously for the two subsequent reactions.

### SEM and EDS analysis

After the S-wave velocity measurement, the dismantled sand layer model was completely dried and coarsely crushed. Selected clods of sand were observed using Scanning Electron Microscopic (SEM) imaging (JSM-7800F, JEOL) to identify the mineral types and precipitation patterns at the microscale, and the chemical composition of precipitates was obtained using energy dispersive X-ray spectroscopy (EDS) analysis.

### Feasibility tests of the soybean crude urease for the cost-efficient EICP treatment

The high cost of lab-grade purified urease may practically limit the field application of the EICP technique. Urease extraction from alternative sources such as watermelon seed and soybean has been investigated in previous studies^39,53–55^. This study adopted the lower cost soybean crude urease for ${\text{SO}}_{4}^{2 - }$ containment. The supernatant solution extracted by centrifuging the mixture of raw soybean powder at 3000 rpm for 15 min was the “soybean crude urease” without purification. Prior to the experiment, urease activity was measured based on the electrical conductivity (EC) change in 1 M urea as described by Whiffin et al.^56^. The urease enzyme hydrolyzes urea to carbonate and ammonium, increasing the EC. The test proceeded to add 3 mL of soybean crude urease into the 27 mL of a solution containing 1.11 M urea to make a standard testing condition (1 M urea), in which the increased rate of EC (mS/cm/min) corresponds to urease activity (11 mM urea/min). The EC was measured at 1-min intervals for 15 min, and then the average increased rate of EC was used for determining the urease activity. Seven concentrations of soybean crude urease, from 5 to 75 g/L with 10 g/L intervals, were prepared. For each soybean concentration, the precipitated CaCO_3_ content was measured from the reaction test on 1.5 M urea:1 M CaCl_2_ solution allowing 3 days of curing time. According to the reaction test result, a specific soybean concentration producing similar CaCO_3_ contents to 0.3 g/L of purified urease was determined to observe the difference in ${\text{SO}}_{4}^{2 - }$ removal efficiency induced by lab-grade purified and soybean crude urease. The measurement of CaCO_3_ contents and evaluation of ${\text{SO}}_{4}^{2 - }$ removal efficiency in the EICP followed the same procedure described in “Determination of EICP recipe for CaCO_3_ formation and${\text{SO}}_{4}^{2 - }$removal: batch test” section. The change of S-wave velocity on the sand specimen by soybean crude urease-triggered EICP treatment and ${\text{SO}}_{4}^{2 - }$ exposure was also monitored with the same experimental setup as in “Monitoring of shear wave velocity in sand layer during EICP reaction and${\text{SO}}_{4}^{2 - }$exposure” section.

## Results and discussion

### Determination of EICP recipe for CaCO_3_ formation and ${\text{SO}}_{4}^{2 - }$ removal

**Figure 4:**
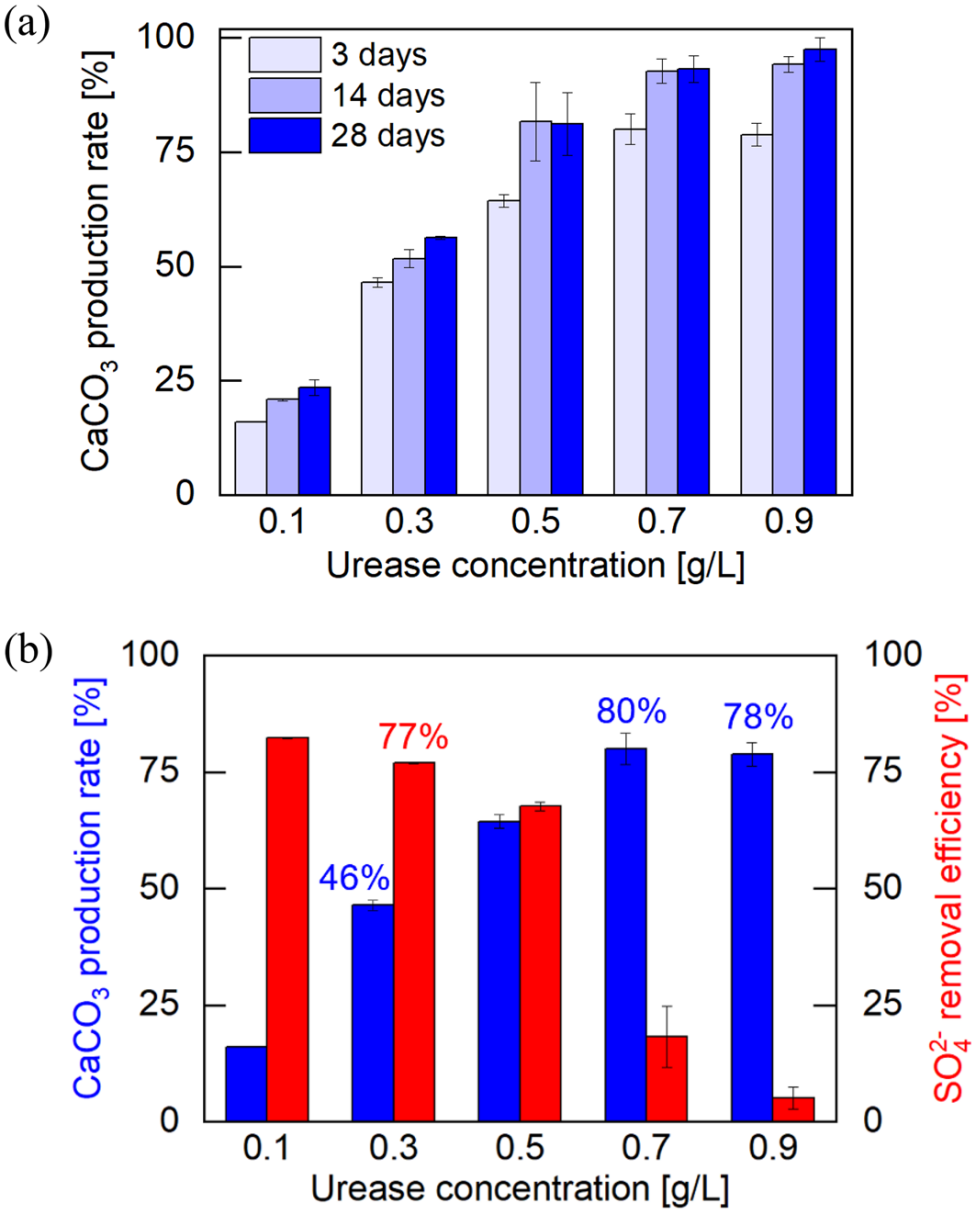
(**a**) CaCO_3_ mineral production rate in uniform EICP substrate with different curing times (3 days, 14 days, and 28 days). (**b**) CaCO_3_ mineral production rate from the 1.5 M urea-1 M CaCl_2_ EICP solution with varied purified urease concentrations after 72 h of curing time (blue bars) and the ${\text{SO}}_{4}^{2 - }$ removal efficiency when 72 h reacted EICP solution was reacted with 0.1 M ${\text{SO}}_{4}^{2 - }$ solution for 24 h (red bars).

The EICP reaction and ${\text{SO}}_{4}^{2 - }$ removal results with solution information are listed in Supplementary Table S1 online. Figure 4a shows the production rate of using 1.5 M urea: 1 M CaCl_2_ solution with various purified urease concentrations and up to 28 days of curing time. It was evident that the CaCO_3_ production increased with time regardless of urease concentration. Urease at a concentration of 0.9 g/L resulted in CaCO_3_ production reaching over 97% after 28 days. With the same EICP solution recipe, the production rate of CaCO_3_ and the removal efficiency of ${\text{SO}}_{4}^{2 - }$ were measured at varying concentrations of purified urease after 3 days of curing (Fig. 4b). Here, ${\text{SO}}_{4}^{2 - }$ removal efficiency was defined as the ratio of the molar concentration of removed ${\text{SO}}_{4}^{2 - }$ to the initial molar concentration (0.05 M) because 0.1 M ${\text{SO}}_{4}^{2 - }$ was mixed with the equal volume of the EICP supernatant solution.

As the purified urease concentration increased (i.e., higher urea hydrolysis rate and faster CaCO_3_ precipitation), the CaCO_3_ production rate gradually increased and reached around 80% with 0.7 g/L and 0.9 g/L of purified urease. As the production rate neared 100%, there were fewer excess calcium cations in the remaining solution, therefore the likelihood of gypsum formation (i.e., ${\text{SO}}_{4}^{2 - }$ removal) decreases. It is therefore anticipated that the higher ${\text{SO}}_{4}^{2 - }$ removal efficiency can be achieved with a lower rate of CaCO_3_ production as shown by the red bar in Fig. 4b. The ${\text{SO}}_{4}^{2 - }$ removal efficiency decreased with higher concentrations of urease solution, exhibiting a trend opposite to that of the CaCO_3_ production rate.

Given the evolution of both production and removal results, a urease concentration of 0.3 g/L (i.e., 46% CaCO_3_ production and 77% ${\text{SO}}_{4}^{2 - }$ removal) was selected to ensure that CaCO_3_ production in the sand layer will exceed 50%, and 50% of calcium cations provided would be retained.

### Evolution of shear stiffness in sand layer during EICP reaction and ${\text{SO}}_{4}^{2 - }$ exposure

**Figure 5:**
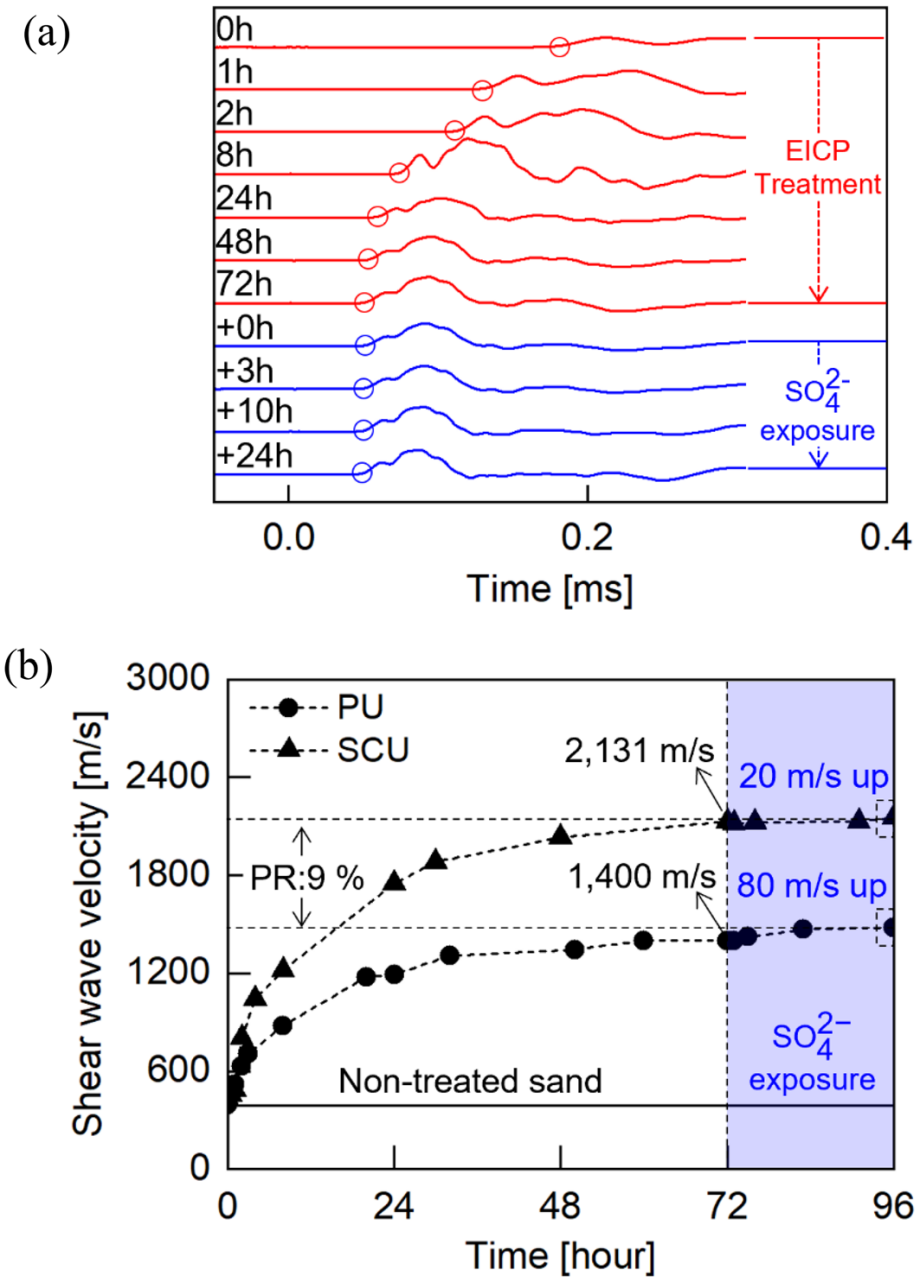
(**a**) Monitored S-waveforms and (**b**) S-wave velocity during the purified urease (PU) and soybean crude urease (SCU) triggered EICP treatment for 72 h and ${\text{SO}}_{4}^{2 - }$ solution exposure for 24 h afterward. *PR = Production rate of CaCO_3_.

The continuously measured waveforms and computed S-wave velocity values were plotted in Fig. 5. The first arrival time of wave traces was marked by hollow circles (Fig. 5a). The EICP reaction with 1.5 M urea, 1 M CaCl_2_, and 0.3 g/L of purified urease was allowed to proceed for the first 72 h, followed by 24 h of ${\text{SO}}_{4}^{2 - }$ solution exposure. In Fig. 5b, as the EICP reaction continued, the S-wave velocity (circular points) sharply increased owing to the inter-particle bonding originating from precipitated carbonate^50,57^ and tended to converge toward the constant velocity of 1400 m/s at 72 h. The S-wave velocity further increased up to 1480 m/s during the exposure to ${\text{SO}}_{4}^{2 - }$ solution implying that the injected ${\text{SO}}_{4}^{2 - }$ reacted with the excess calcium cations to form gypsum. It is noted that the shear stiffness (G) is proportional to the square of the S-wave velocity (i.e., G = ρ⋅V_s_^2^). Therefore, the increase of S-wave velocity from 390 to 1400 m/s indicated 13 times increase in the strengthening of the embedded sand layer for shear stiffness during carbonate precipitation and 1.12 times further enhancement by gypsum precipitation. The precipitated CaCO_3_ weight fraction of this sample was measured as 1.61% by using acid digestion. The increase in S-wave velocity after the EICP treatment and ${\text{SO}}_{4}^{2 - }$ solution exposure appeared similar to former related studies where the S-wave velocities after bio-cementation treatment in the sand columns were over one–sixfold than the original status with 1–2 w% CaCO_3_ content^50,57^. The effects of gypsum precipitation within soil were examined in previous studies. Dejong et al.^58^ showed 2 times increase in S-wave velocity by 5 w% gypsum cementations. Lee et al.^59^ mentioned that the shear modulus more increased as the weight percent of gypsum with sand increased. Although the theoretical estimation of gypsum precipitation was nominal (0.22w%) in this test, it obviously contributed to the increase in S-wave velocity.

The concentration of ${\text{SO}}_{4}^{2 - }$ in the effluent from this specimen was measured using ion chromatography analysis mentioned in “Determination of EICP recipe for CaCO_3_ formation and${\text{SO}}_{4}^{2 - }$removal: batch test” section. The effluent showed 20% ${\text{SO}}_{4}^{2 - }$ removal efficiency, which was 57% lower than the liquid test result in “Determination of EICP recipe for CaCO_3_ formation and${\text{SO}}_{4}^{2 - }$removal” section. Note that the originally proposed concept of the liner system consisted of upper clay-middle partially EICP treated sand-lower clay through which the invaded landfill leachate may pass through the upper clay layer with very lower hydraulic conductivity (e.g., < 1 × 10^–7^ cm/s of clay) and stay long enough within the sand layer for CaCO_3_ and gypsum precipitation due to the lower clay base liner. In this oedometer experiment, only Junmunjin sand was tested to demonstrate the function of the sand layer as ${\text{SO}}_{4}^{2 - }$ containment, and the pre-existing EICP reactive solution in the sand layer located in the oedometer cell was supposed to be expelled out of the cell simultaneously as 0.1 M Na_2_SO_4_ solution from the reservoir (Fig. 3) was continuously circulated for 26 min in the closed system. It is noted that the measured permeability of the fully treated bio-cemented sand was 2.2 × 10^–2^ cm/s that was slightly lower than that of the non-treated sand (6.2 × 10^–2^ cm/s). Therefore, the immediate gypsum precipitation might occur throughout the entire connected system including the oedometer cell, reservoir, and connecting tubes which resulted in the discrepancy of ${\text{SO}}_{4}^{2 - }$ removal efficiencies between the batch test in “Determination of EICP recipe for CaCO_3_ formation and${\text{SO}}_{4}^{2 - }$removal” section and the oedometer circulation test in 3.2 (respectively 77% and 20%).

Nevertheless, these results imply that the retention of excess calcium cations as a byproduct of EICP can not only increase the geomechanical resistance of the sand layer but also function as a potential containment reservoir for ${\text{SO}}_{4}^{2 - }$.

### Visual inspection of CaCO_3_ and gypsum formation

**Figure 6:**
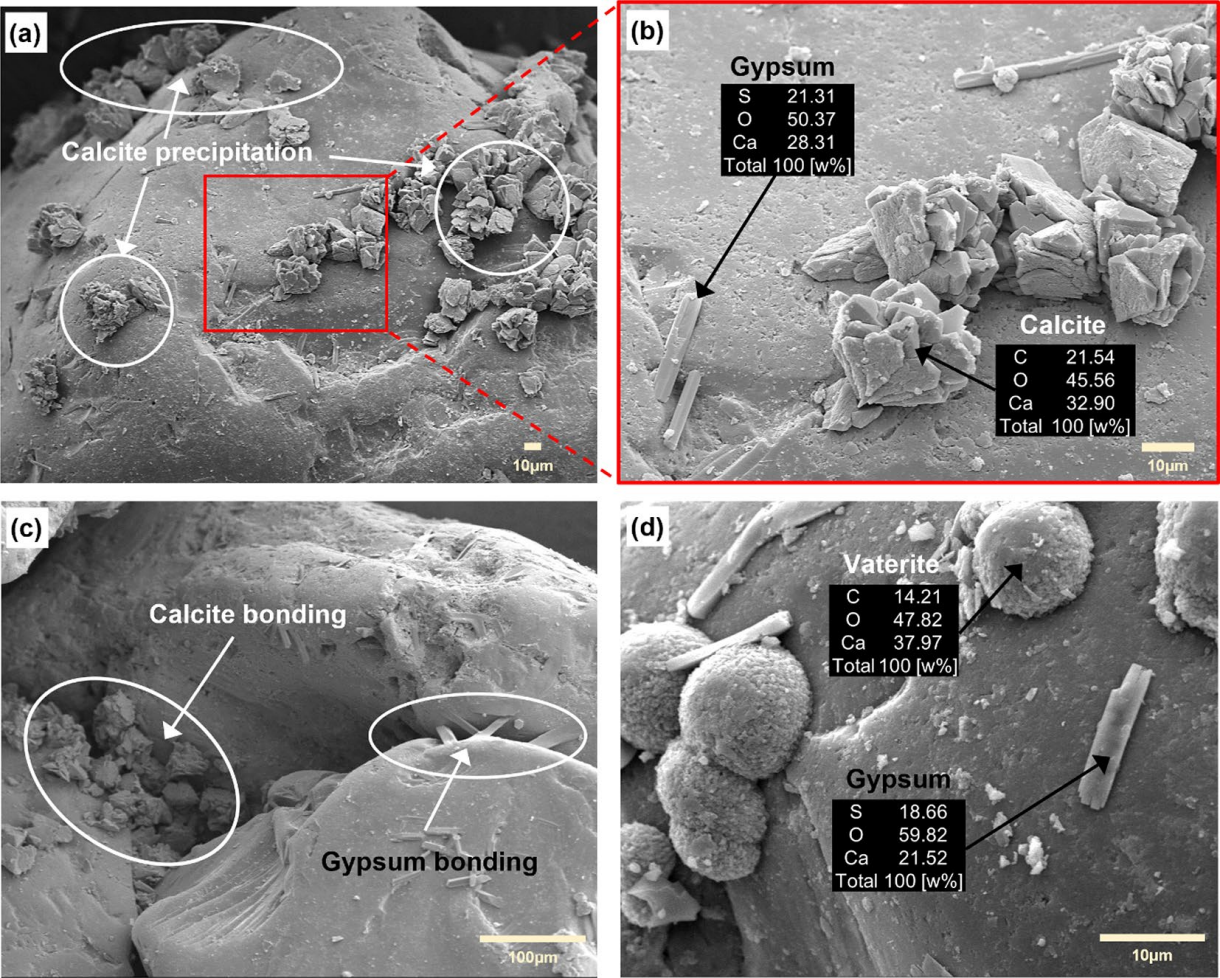
SEM images and energy-dispersive X-ray spectroscopy (EDS) analysis of the specimen after ${\text{SO}}_{4}^{2 - }$ solution exposure through the partially EICP-treated sand sample promoted by purified urease (**a**–**c**) and by soybean crude urease (**d**).

The SEM images taken for recovered samples showed that the precipitated carbonates had a rhombohedral shape that is representative of the most stable CaCO_3_ crystal configuration (Fig. 6a–c). Relatively large calcite minerals (over 30 µm) were nonuniformly scattered on the sand particle surface and closely located to each other presumably due to high affinity to the new nucleation of dissolved calcium and carbonate ions for further crystal growth (Fig. 6a). Needle-like narrow crystals, commonly observed in gypsum cementation^58^, were identified in the enlarged image (Fig. 6b). Both calcite and gypsum were chemically analyzed using EDS and the target minerals were successfully identified by confirming the weight fraction of elemental components such as carbon (C), oxygen (O), calcium (Ca), and sulfur (S). Figure 6c shows that these calcite and gypsum minerals contributed to enhancing the shear stiffness as previously described in the literature^50,58^. Gypsum minerals were precipitated by the reaction of calcium cations with circulated ${\text{SO}}_{4}^{2 - }$, while their existence was not extensively observed because of the limited source of excess calcium ions in the solution. Despite its nominal appearance, 77% of ${\text{SO}}_{4}^{2 - }$ was expected to be removed based on the controlled experiment as shown in Fig. 4.

### ${\text{SO}}_{4}^{2 - }$ removal efficiency on soybean crude urease-triggered EICP

**Figure 7:**
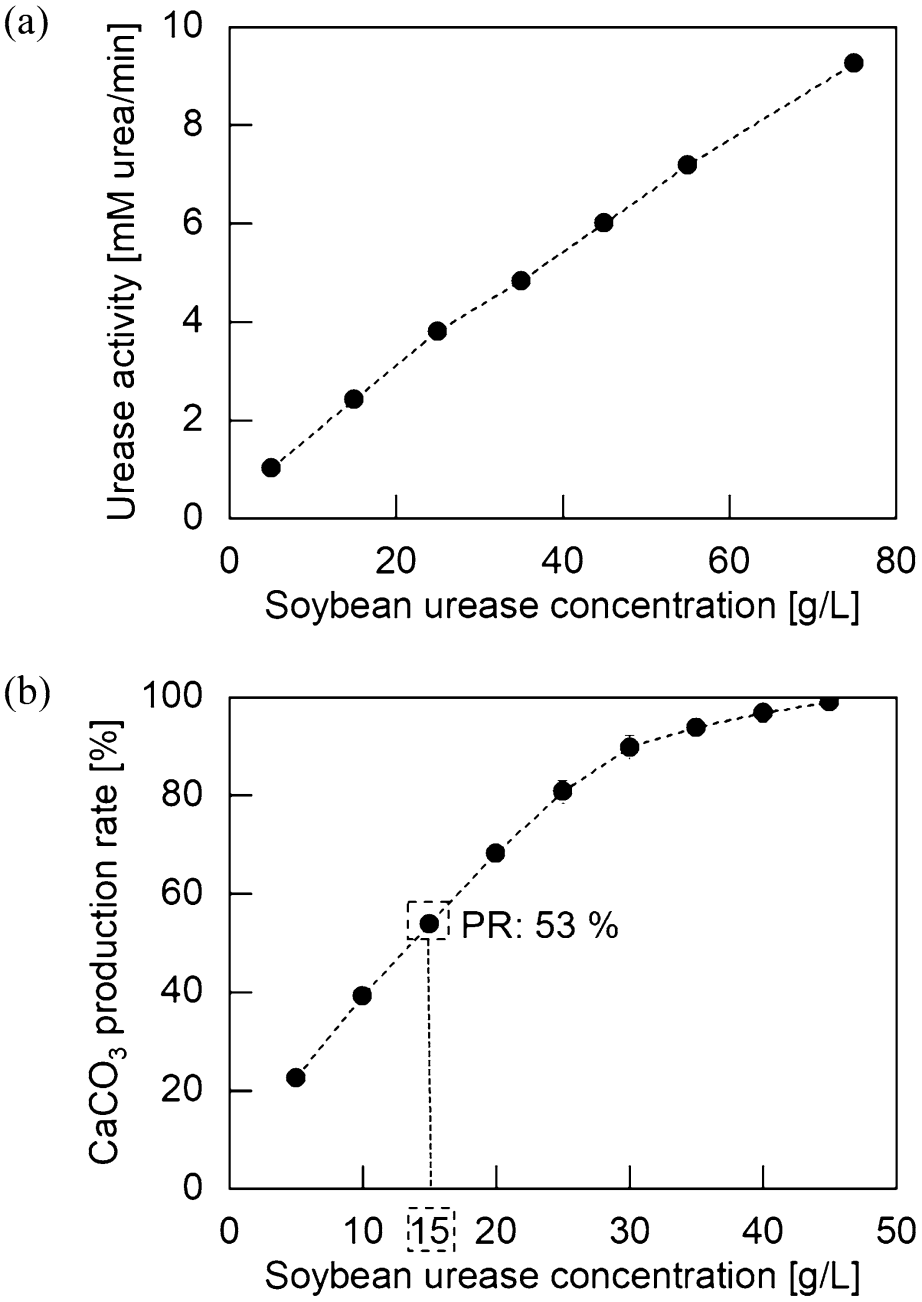
Characterization of soybean crude urease. (**a**) Activity measurement (**b**) CaCO_3_ production rate after 72 h of EICP treatment with 1 M CaCl_2_ and 1.5 M urea at varied soybean urease concentrations.

Figure 7 shows the characterization of soybean crude urease used in this study. The activity linearly increased as the soybean concentration increased with the range of 1.02–9.26 mM urea/min (Fig. 7a). Previous studies demonstrated that the activity of bacteria or urease from 0.5 to 10 mM urea/min was suitable for soil treatment; therefore, the soybean concentrations (i.e., 5–75 g/L) used in this study were appropriate for EICP treatment^56,60^. In Fig. 7b, the corresponding production rate of CaCO_3_ by different soybean concentrations exhibited a linear increase up to a soybean concentration of 25 g/L and gradually converged towards 100% at 45 g/L when the test was conducted according to the methods detailed in “Monitoring of shear wave velocity in sand layer during EICP reaction and${\text{SO}}_{4}^{2 - }$exposure” section. Revisiting Fig. 4, where 0.3 g/L of purified urease was selected to produce 46% of CaCO_3_, 15 g/L of soybean crude urease was selected to examine the efficiency of both CaCO_3_ precipitation and gypsum formation.

The EICP triggered by 15 g/L of soybean crude urease showed 18% of ${\text{SO}}_{4}^{2 - }$ removal, which was 59% lower than the test using 0.3 g/L of purified urease. Revisiting Fig. 5b, the measurement of S-wave velocity (triangular points) indicated that the wave velocity increased from 430 to 2031 m/s for 72 h of the EICP curing period, showing 1.85% of the precipitated CaCO_3_ weight fraction, and was followed by a trivial increase of approximately 20 m/s during the subsequent 24 h of ${\text{SO}}_{4}^{2 - }$ exposure. During two subsequent reactions, the significant difference in wave velocity between purified and soybean crude urease-triggered EICP was caused by the difference in the amount of CaCO_3_ present (i.e., 9%).

In addition, the results for ${\text{SO}}_{4}^{2 - }$ removal efficiency of the soybean crude urease in the effluent showed effectiveness at 20% lower than the 0.3 g/L of purified urease under the same measurement conditions. Revisiting Fig. 6d, spherically shaped vaterite and gypsum crystals were confirmed while the amount of gypsum observed was much smaller. Considering that soybean crude urease includes excess protein and numerous tiny suspended particles, it is natural for vaterite minerals to be stabilized in the EICP because these factors retard the dissolution and recrystallization into calcite, absorbing into the crystal surface and changing the surface energy^61–63^. The noticeable difference between the removal efficiency from both enzymes can be also explained by impurities in the soybean crude urease. In general, crystallization processes are interrupted by additives, which retard induction times and prohibit further crystal growth beyond the critical nucleus size^64–66^. The impurities associated with the soybean crude urease can act as a nucleation inhibitor, while also decreasing ${\text{SO}}_{4}^{2 - }$ removal efficiency.

In summary, despite the contribution of shear stiffness improvement by CaCO_3_ and lower price, the soybean crude urease had a lower efficiency of ${\text{SO}}_{4}^{2 - }$ containment.

### Effect of gypsum powder on ${\text{SO}}_{4}^{2 - }$ removal efficiency

In order to improve the ${\text{SO}}_{4}^{2 - }$ removal ability, nucleation seeds in the form of high purity gypsum powder (i.e., 0.6 M CaCl_2_ and 0.6 M Na_2_SO_4_) were preemptively added to the 15 g/L crude urease-EICP solution before the ${\text{SO}}_{4}^{2 - }$ reaction. The effect of dosage differences and reaction time on dissolved ${\text{SO}}_{4}^{2 - }$ removal was investigated with concentrations of 4 and 6 g/L of gypsum powder and 24 and 72 h reaction time. The EICP reaction was allowed to proceed for 72 h, then an equivalent volume of 0.1 M ${\text{SO}}_{4}^{2 - }$ solution was added to the reacted batch without extracting EICP supernatant. After 6 days reaction time (3 days EICP + 3 days ${\text{SO}}_{4}^{2 - }$ reaction), CaCO_3_ contents were measured.

**Figure 8:**
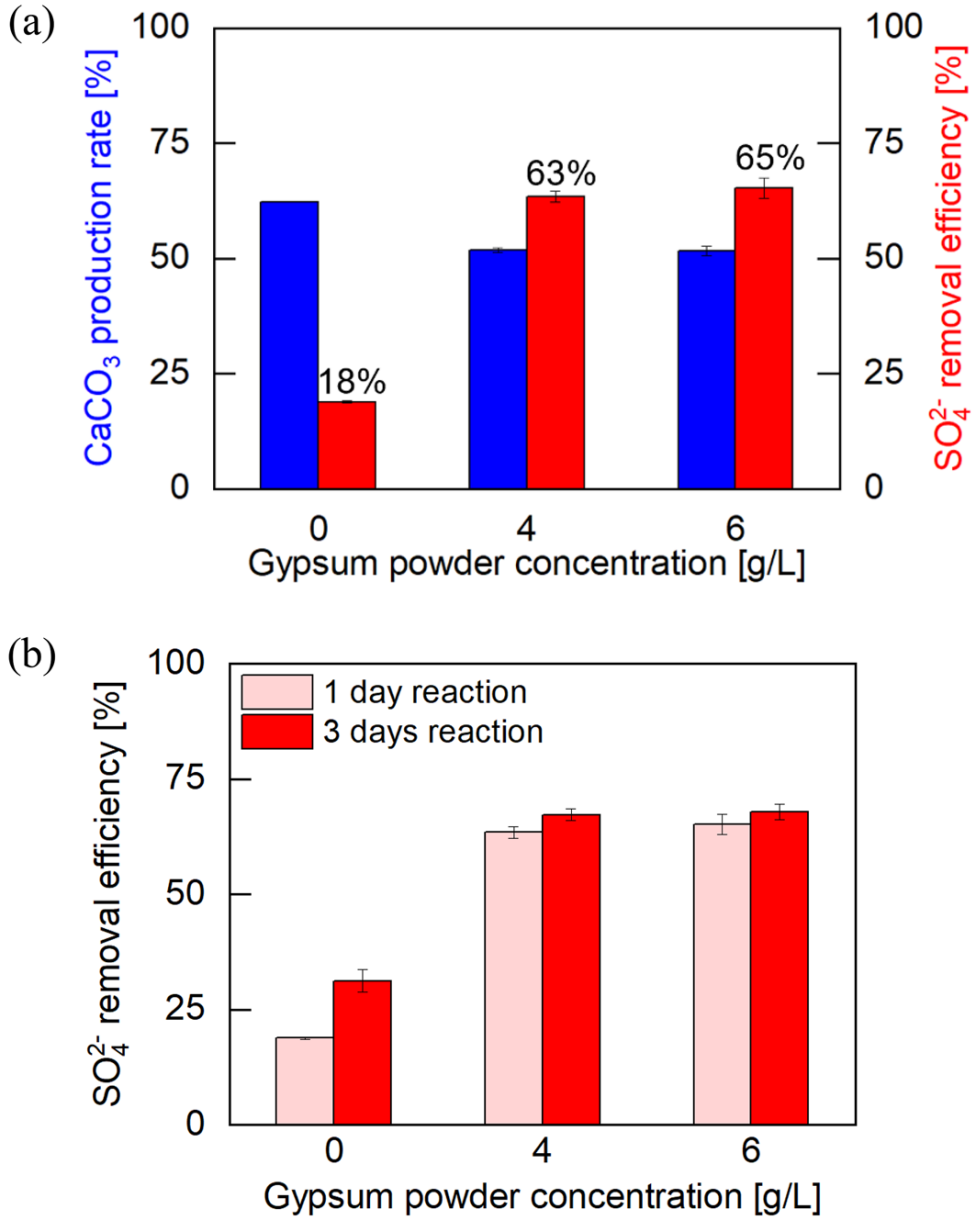
Test results with added gypsum powder. (**a**) ${\text{SO}}_{4}^{2 - }$ removal efficiency and CaCO_3_ production rate within the crude urease triggered EICP solution. CaCO_3_ contents were obtained after 6 days (EICP 3 days + reaction 3 days) and ${\text{SO}}_{4}^{2 - }$ removal efficiency was measured after 1 day ${\text{SO}}_{4}^{2 - }$ reaction. (**b**) ${\text{SO}}_{4}^{2 - }$ removal efficiency with increasing ${\text{SO}}_{4}^{2 - }$ reaction time.

Figure 8a shows the CaCO_3_ production rate (blue bar) and the ${\text{SO}}_{4}^{2 - }$ removal efficiency (red bar) using different concentrations of gypsum powder. The CaCO_3_ production rate decreased by approximately 10% after the addition of gypsum powder. This suggests that the gypsum powder particles inhibited the binding of Ca^2+^ and ${\text{CO}}_{3}^{2 - }$ ions resulting in a lower CaCO_3_ production rate. However, the ${\text{SO}}_{4}^{2 - }$ removal efficiency increased by 45% and 47% after adding 4 g/L and 6 g/L of gypsum powder, respectively. Gypsum powder provides a favorable environment for gypsum formation, providing additional sites for gypsum crystals to form in which molecules can be readily assembled^67^. Figure 8b shows the ${\text{SO}}_{4}^{2 - }$ removal efficiency with increasing reaction time. While the sample containing no gypsum powder showed increased removal efficiency with increased reaction time, both gypsum powder samples exhibited no significant difference at the 1 and 3 days time points, indicating that gypsum powder reduced the crystallization rate. The detailed test results are summarized in Supplementary Table S2 online.

### Economical aspect of proposed method in field application

The total cost was compared for three cases: (1) purified urease (PU), (2) soybean crude urease (SCU), and (3) soybean crude urease with gypsum powder (SCU + G). These cost values were calculated for the unit volume of Jumunjin sand with a relative density of 70% and the details were shown in Table 2. Note that the cost of gypsum powder was derived by considering the price of lab-grade CaCl_2·_2H_2_O and Na_2_SO_4_ corresponding to 4 g/L concentration. For 1-m^3^ soil treatment by PU, the cost was calculated at $15,386 USD while the cost of SCU amounted to only $50 USD (300 times lower). The cost of SCU + G was $8900 USD, because of the high costs associated with the chemical agents used to produce the gypsum powder. Lower-cost gypsum such as flue gas desulfurization gypsum, a type of waste gypsum, may be considered as a material potentially usable for $${\text{SO}}_{4}^{2 - }$$ removal due to its major chemical composition (CaSO_4_), alkalinity characteristic (pH 8–10) similar with a favorable pH range of the EICP reaction (pH 8–9), and lower cost of approximately 4.24 USD/ton^68,69^.

**Table 2:** Estimated cost of each urease enzyme and gypsum powder to treat 1-m^3^ of Jumunjin sand.

Case	Component	Price (USD/g)	Concentration (g/L)	Usage per 1-m^3^ of Jumunjin sand (g)	Total cost (USD)
PU	Purified urease	125.7	0.3	122	15,386
SCU + G	Soybean crude urease (SCU)	0.008	15	6120	8912**
	Gypsum powder (G)	0.407*	4	44,409	

*The price was determined by summing each price of 0.232 M CaCl_2_·2H_2_O and 0.232 M Na_2_SO_4_ which corresponds to 4 g of gypsum in the 1 L solution.

**Total cost of SCU + G (8912 USD) = 49 USD (SCU) + 8863 USD (G).

## Conclusions

This study explored the feasibility of manipulation of EICP substrate composition to provide excess calcium cations for effective ${\text{SO}}_{4}^{2 - }$ removal. Lab-grade purified urease and low-cost soybean crude urease were used to examine the ${\text{SO}}_{4}^{2 - }$ removal efficiency of the substrate. For a 1.5 M urea:1 M CaCl_2_ EICP solution, a 0.3 g/L concentration of purified urease enzyme was selected to consume half of the provided calcium cations for CaCO_3_ precipitation for 72 h of curing, showing 77% ${\text{SO}}_{4}^{2 - }$ removal efficiency. The shear stiffness of sand increased about 13 times during EICP treatment and increased again 1.12 times after the subsequent formation of gypsum. When the soybean crude urease was used for the EICP reaction, ${\text{SO}}_{4}^{2 - }$ removal efficiency in the batch test was 59% lower than purified urease-EICP reaction due to the impurities in the solution, showing only nominal formation of gypsum in the bio-cemented sand. This limitation was overcome by adding synthetic gypsum powder to the soybean crude urease solution and the ${\text{SO}}_{4}^{2 - }$ removal efficiency increased by 40% with faster reaction times. To lower the operational costs for the practical application, it is recommended to use soybean crude urease as ECIP agents with gypsum powder.

## Supplementary Information

Supplementary Information.

## Data Availability

The data generated and analyzed during this work are included in this paper and available from the corresponding author upon request.
