# Supplementary material for: Containment of sulfate in leachate as gypsum (CaSO4·2H2O) mineral formation in bio-cemented sand via enzyme-induced carbonate precipitation
Source: Sci Rep. 2023 Jul 6;13:10938. doi: 10.1038/s41598-023-37772-z (PMC10326029; doi:10.1038/s41598-023-37772-z)
Supplement: Supplementary file 1 — Supplementary Information. [file 41598_2023_37772_MOESM1_ESM.docx]

**Supplementary Table S1.** Solution information of the EICP treatment using the purified urease for $\mathrm{SO}_{4}^{2-}$ removal. 3 days of curing time on the EICP and 24 hours of reaction with $\mathrm{SO}_{4}^{2-}$ were applied to all samples. *B-4 and **B-5 were cured for 14 and 28 days, respectively.

| Urease  type | Specimen number | Basic EICP compound | | | Precipitated CaCO_3_ [M] | $\mathrm{SO}_{4}^{2-}$ removal efficiency [%] |
| --- | --- | --- | --- | --- | --- | --- |
|  |  | Urea [M] | CaCl_2_ [M] | Urease [g/L] |  |  |
| Purified urease | A-1 | 1.5 | 1 | 0.1 | 0.16 | 82.56 |
| Purified urease | A-2 | 1.5 | 1 | 0.1 | 0.16 | 82.23 |
| Purified urease | A-3 | 1.5 | 1 | 0.1 | 0.16 | 82.38 |
| Purified urease | B-1 | 1.5 | 1 | 0.3 | 0.46 | 77.15 |
| Purified urease | B-2 | 1.5 | 1 | 0.3 | 0.48 | 76.72 |
| Purified urease | B-3 | 1.5 | 1 | 0.3 | 0.46 | 76.98 |
| Purified urease | *B-4 | 1.5 | 1 | 0.3 | 0.50 | 83.0 |
| Purified urease | **B-5 | 1.5 | 1 | 0.3 | 0.56 | 70.40 |
| Purified urease | C-1 | 1.5 | 1 | 0.5 | 0.66 | 67.55 |
| Purified urease | C-2 | 1.5 | 1 | 0.5 | 0.64 | 66.52 |
| Purified urease | C-3 | 1.5 | 1 | 0.5 | 0.63 | 68.84 |
| Purified urease | D-1 | 1.5 | 1 | 0.7 | 0.75 | 9.23 |
| Purified urease | D-2 | 1.5 | 1 | 0.7 | 0.81 | 20.77 |
| Purified urease | D-3 | 1.5 | 1 | 0.7 | 0.83 | 24.77 |
| Purified urease | E-1 | 1.5 | 1 | 0.9 | 0.78 | 7.46 |
| Purified urease | E-2 | 1.5 | 1 | 0.9 | 0.77 | 2.81 |

**Supplementary Table S2.** Solution information of the EICP treatment using the soybean crude urease for $\mathrm{SO}_{4}^{2-}$ removal with different gypsum powder concentrations. 3 days of curing time on the EICP was applied to all samples and the amount of CaCO_3_ obtained after 3 days of reaction process with $\mathrm{SO}_{4}^{2-}$ was measured.

| Urease  type | Specimen number  (Reaction day) | Basic EICP compound | | | Gypsum powder [g/L] | Precipitated CaCO_3_  [M] | $\mathrm{SO}_{4}^{2-}$ removal efficiency [%] |
| --- | --- | --- | --- | --- | --- | --- | --- |
|  |  | Urea [M] | CaCl_2_ [M] | Urease [g/L] |  |  |  |
| Crude urease | C-1  (1 day) | 1.5 | 1 | 15 | 0 | - | 18.85 |
| Crude urease | C-2  (3 days) | 1.5 | 1 | 15 | 0 | 0.62 | 31.22 |
| Crude urease | CA-1  (1 day) | 1.5 | 1 | 15 | 4 | - | 62.92 |
| Crude urease | CA-2  (1 day) | 1.5 | 1 | 15 | 4 | - | 64.85 |
| Crude urease | CA-3  (1 day) | 1.5 | 1 | 15 | 4 | - | 62.59 |
| Crude urease | CB-1  (1 day) | 1.5 | 1 | 15 | 6 | - | 65.71 |
| Crude urease | CB-2  (1 day) | 1.5 | 1 | 15 | 6 | - | 67.20 |
| Crude urease | CB-3  (1 day) | 1.5 | 1 | 15 | 6 | - | 62.89 |
| Crude urease | CA-4  (3 days) | 1.5 | 1 | 15 | 4 | 0.51 | 66.11 |
| Crude urease | CA-5  (3 days) | 1.5 | 1 | 15 | 4 | 0.53 | 67.15 |
| Crude urease | CA-6  (3 days) | 1.5 | 1 | 15 | 4 | 0.51 | 68.61 |
| Crude urease | CB-4  (3 days) | 1.5 | 1 | 15 | 6 | 0.52 | 68.63 |
| Crude urease | CB-5  (3 days) | 1.5 | 1 | 15 | 6 | 0.50 | 69.16 |
| Crude urease | CB-6  (3 days) | 1.5 | 1 | 15 | 6 | 0.53 | 66.04 |
